# Palmitic Acid Induces Inflammatory Environment and Is Involved in Pyroptosis in a Human Dental Pulp Cell Line

**DOI:** 10.3390/dj14010051

**Published:** 2026-01-12

**Authors:** Takashi Muramatsu, Akihide Yanagisawa, Keisuke Mitomo, Kana Takada, Masahiro Furusawa, Yoshihiro Abiko, Han-Sung Jung

**Affiliations:** 1 Department of Operative Dentistry, Cariology and Pulp Biology, Tokyo Dental College, Tokyo 101-0061, Japan; 2 Department of Endodontics, Tokyo Dental College, Tokyo 101-0061, Japan; 3 Takada Dental Office, Tokyo 179-0072, Japan; 4 Division of Oral Medicine and Pathology, Department of Human Biology and Pathophysiology, School of Dentistry, Health Sciences University of Hokkaido, Hokkaido 061-0293, Japan; yoshi-ab@hoku-iryo-u.ac.jp; 5 Division in Anatomy and Developmental Biology, Department of Oral Biology, Taste Research Center, Oral Science Research Center, BK 21 FOUR Project, Yonsei University College of Dentistry, Seoul 03722, Republic of Korea; hsjung@yuhs.ac

**Keywords:** palmitic acid, dental pulp, inflammatory cytokine, hyperlipidemia, diabetes mellitus, lipopolysaccharide, pyroptosis

## Abstract

**Background/Objectives**: We investigated whether palmitic acid (PA) induced the expression of inflammatory cytokines and was involved in pyroptosis in a human dental pulp cell line. **Methods**: Human dental pulp cells cultured in Minimum Essential Medium Alpha (αMEM) were treated with 1 µg/mL LPS and/or PA (100, 300 and 500 µM). As a control, αMEM was added in the culture medium. The WST-1 assay was performed to assess cell proliferation, and morphological changes in cells were examined. RNA expression of *IL-1β*, *IL-6*, *TNF-α*, *caspase-4* and *gasdermin d* were detected by quantitative RT-PCR (qPCR). **Results**: The WST-1 assay showed that cell viability decreased by 36% at 300 µM and 47% at 500 µM PA compared to the control (*p* < 0.05). Cell morphology revealed slight shrinkage in 100, 300 and 500 µM PA groups. RNA expression of *IL-1β* and *IL-6* in the PA groups was significantly higher than that in the control groups (*p* < 0.05), while RNA expression of *TNF-α* in the PA group was the same as that of control group. The mRNA expression of *caspase-4* and *gasdermin d* in PA groups was significantly higher than that in control group (*p* < 0.05). Likewise, the concentration of IL-1β and IL-6 was significantly higher in both LPS and PA groups than that in the LPS or PA groups (*p* < 0.05). **Conclusions**: The results of this study suggest that PA induces the expression of inflammatory cytokines and is involved in pyroptosis in a human dental pulp cell line.

## 1. Introduction

Relationships between oral infections and systemic diseases, such as diabetes mellitus and hyperlipidemia, have been studied for over 20 years. The association between periodontitis and systemic diseases has been elucidated, and “periodontal medicine” is established as an area of periodontal research [1,2]. Metabolic diseases affect periodontal disease, and people with obesity and type 2 diabetes mellitus are more likely to have periodontitis [3,4,5]. On the other hand, the association between pulp lesions and systemic diseases remains unknown. Only a few human histologic pulp studies have been reported in patients with diabetes about vascular changes [6,7], calcification [7] and odontalgia [8]. Although the mechanism of calcification is unclear, it is speculated that asymptomatic pulp inflammation leads to cell death, followed by dystrophic calcification in the patients with diabetes.

Hyperlipidemia, one of major metabolic disorders, is known to be induced by obesity and type 2 diabetes mellitus [9,10]. Interestingly, hyperlipidemia using model mice is also involved in the higher expression of inflammatory cytokines such as IL-6 and TNF-α and alveolar bone loss [11,12], and therefore, hyperlipidemia would be a risk factor of inflammation in oral cavity [13]. With regard to dental pulp tissue, hyperlipidemia enhances pulp stone formation in humans [14], and delays dentin mineralization in mouse incisors [15]. On the other hand, increased levels of plasma free fatty acid (FFA), which is associated with hyperlipidemia, induce inflammatory responses via toll-like receptor (TLR) signaling [16,17]. Patients with diabetes are often accompanied by hyperlipidemia, and therefore, dental pulp lesions such as vascular changes, calcification and odontalgia may be related to hyperlipidemia. However, detailed relevance between hyperlipidemia and dental pulp inflammation remains unknown.

Palmitic acid (PA) is the predominant saturated FFA in dietary fats and in human blood [18]. Increased blood levels of PA are observed in patients with excess fat intake, obesity and insulin-resistant diabetes [19,20]. PA has been reported to induce inflammation [21,22,23] and pyroptosis [24] in periodontal tissues. However, it remains unknown whether PA can induce inflammation and pyroptosis in dental pulp.

Pyroptosis is a kind of cell death induced by inflammation. There are two pathways that initiate pyroptosis: the caspase-1-mediated canonical pathway and the caspase-4/5/11-mediated noncanonical pathway [25]. In the canonical pathway, upon stimuli, inflammasomes including NOD-like receptor protein 3 (NLRP3) assemble to activate caspase-1 that cleaves pro-IL-1β, pro-IL-18 and gasdermin d into their mature forms. In response to inflammatory caspases, N-terminal of gasdermin d, a downstream effector of pyroptosis, can be activated and form pores in the cell membrane [26], resulting in the extracellular release of mature IL-1β, cell swelling and eventually cell death [27]. In the non-canonical pyroptosis pathway, lipopolysaccharide (LPS) is the immediate activator of human inflammatory caspase-4/caspase-5 or murine caspase-11, which directly cleaves gasdermin d and initiates pyroptosis [27,28]. The levels of pyroptosis-related proteins including NLRP3, caspase-1, caspase-4 and IL-18, were increased in inflammatory periodontal tissue compared to healthy periodontal tissue of human and rat [29]. Recently, pyroptosis has been reported to play an important role in obesity- and diabetes-associated inflammation [30]. However, whether PA can induce pyroptosis in dental pulp remains unknown.

From the background of the earlier studies, we hypothesized that PA induces inflammation and pyroptosis in dental pulp cells. The aim of this study is to investigate the expression of inflammatory cytokines and pyroptosis by PA in human dental pulp cells.

## 2. Materials and Methods

### 2.1. Reagents

PA was obtained from Sigma-Aldrich (P0500, Tokyo, Japan). Minimum Essential Medium Alpha (αMEM) and penicillin-streptomycin were purchased from Thermo Fisher Scientific Inc. (12571063, 15140122, Waltham, MA, USA). Fetal bovine serum (FBS) was obtained from Biosera (FB-1003, Kansas City, MO, USA). *Porphyromonas gingivalis*-derived LPS was purchased from InvivoGen (tlrl-pglps, San Diego, CA, USA).

### 2.2. Cell Culture

**Figure 1:**
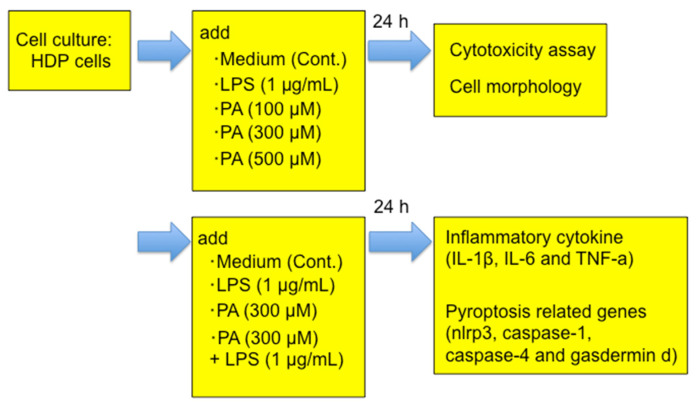
A flowchart of the experiment.

We used human dental pulp cells (HDPs) immortalized by transfection with a human telomerase transcriptase gene. The cells were provided by Professor Takashi Takata (Hiroshima University Graduate School, Hiroshima, Japan; Shunan University, Shunan, Japan). HDP characteristics were previously described [31]. HDPs were cultured in αMEM supplemented with 10% FBS and 100 U/mL penicillin-streptomycin at 37 °C in a humidified atmosphere of 95% air and 5% CO_2_. The use of the HDP was approved by the Ethics Committee of Tokyo Dental College (No.1025). A flowchart of the experiment is shown in Figure 1.

### 2.3. In Vitro Cytotoxicity Assay

Cytotoxicity was examined using the WST-1 assay (501594, Roche Diagnostics, Basel, Switzerland). HDPs were seeded at 1 × 10^4^ cells per well on 96-well plates in αMEM and cultured for 24 h. Cells were treated with 100, 300 and 500 µM PA, and 1 µg/mL *P. gingivalis*-derived LPS. As a control, αMEM was added in the culture medium. After incubation for 24 h, WST-1 reagent was added to each well and incubated for 1 h. The colorimetric reaction was then stopped and assessed using a microplate reader (Molecular Devices, Sunnyvale, CA, USA) at 450 nm. Seven samples were used, and each sample was investigated in triplicate.

### 2.4. Cell Morphology

Twenty-four hours after the addition of PA, HDPs were fixed in 4% paraformaldehyde solution (30525-89-4, Fujifilm-Wako Pure Chemical Corporation, Osaka, Japan) for two hours. After washing three times in phosphate-buffered saline (PBS) for 5 min each, HDPs were observed under a microscope (Axiophot 2; Carl Zeiss, Oberkochen, Germany) to confirm cell viability and morphology. Cell appearance was randomly documented with photographs.

### 2.5. Quantitative Reverse-Transcriptase Polymerase Chain Reaction (qPCR)

Total RNA was extracted from HDPs (1 × 10^5^ cells) using the acid guanidinium-thiocyanate-phenol-chloroform (AGPC) method with TRIzol (15596026, Invitrogen, Grand Island, NY, USA) and the synthesis of complementary DNA (cDNA) was performed as previously described [32,33]. Total RNA (1 μg) was reverse-transcribed into cDNA using the High-Capacity cDNA Reverse Transcription Kit (4368814, Applied Biosystems, Foster City, CA, USA). qPCR analyses were performed with the ABI7500 Fast System (Applied Biosystems) using an equal volume of TaqMan Fast Universal PCR Master Mix No AmpErase reagent (4352042, Applied Biosystems). TaqMan probes of human target genes were *IL-1β* (Hs01555410_m1), *IL-6* (Hs00985639_m1), *TNF-α* (Hs01113624_g1), *nlrp3* (Hs00918082_m1), *caspase-1* (Hs01030829_m1), *caspase-4* (Hs01031951_m1) and *gasdermin d* (Hs00986748_g1) with *GAPDH* as the endogenous control. Reaction conditions consisted of 40 cycles of PCR comprising denaturation at 95 °C for 3 s and annealing and extension at 60 °C for 30 s. The relative expression of the genes of interest was estimated using the ΔΔ threshold cycle (Ct) method [34]. Briefly, *GAPDH* was used to normalize the amount of target gene messenger RNA (mRNA). Its Ct value was subtracted from that of the target gene to obtain a ΔCt value. The difference (ΔΔCt) between the ΔCt values of samples for the target genes and that of the calibrator was assessed. Five to seven samples per each group were used, and the qPCR was performed in triplicate.

### 2.6. Enzyme-Linked Immunosorbent Assay (ELISA)

Culture media was collected from each group after the addition of LPS and/or palmitate. The levels of IL-1β, IL-6 and TNF-α were detected by biotinylated anti-human monoclonal antibodies using ELISA kits according to the manufacturer’s instructions (DLB50, D6050, DTA00D; R&D Systems Inc, Minneapolis, MN, USA). The absorbance was detected using a microplate reader (Molecular Devices) at 450 nm. The levels of IL-1β, IL-6 and TNF-α were summarized from triplicate measurements.

### 2.7. Statistical Analysis

Quantitative data were expressed as the means ± standard deviation (SD). Analysis software (IBM SPSS 27.0 J for Windows; SPSS Japan, Tokyo, Japan) was used for statistical processing. The significance of differences among groups was assessed using a one-way analysis of variance (ANOVA) followed by Bonferroni multiple comparison tests, with *p* < 0.05 being significant.

## 3. Results

### 3.1. Cell Viability

**Figure 2:**
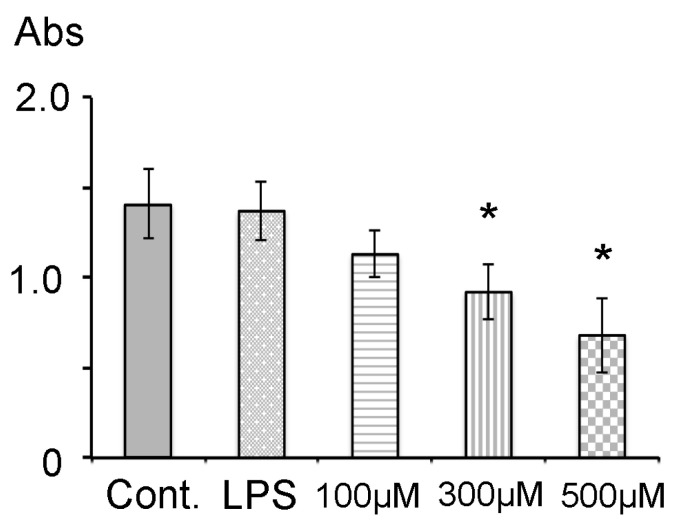
Cytotoxic assay. Data were expressed as the means ± standard deviation (SD) (n = 7 each). The cytotoxicities of 1 µg/mL LPS, 100, 300 and 500 µM of PA were estimated in HDPs using the WST-1 assay. Cont.: control group; * *p* < 0.05.

The viability of HDPs following the addition of 1 µg/mL LPS, 100, 300 and 500 µM PA was investigated (n = 7 each group). The results of the WST-1 assay showed that absorbance ranged between 0.68 and 1.41. The absorbance in PA group was decreased in a dose-dependent manner, and cell viability decreased by 36% at 300 µM and 47% at 500 µM PA compared to the control (*p* < 0.05). Statistically significant differences were seen between the control group and 300 µM group, and between the control group and 500 µM group (Figure 2). Furthermore, there were statistical differences among LPS group, 300 µM group and 500 µM group. No significant differences were detected among the control group, LPS group and 100 µM group.

### 3.2. Cell Morphology

**Figure 3:**
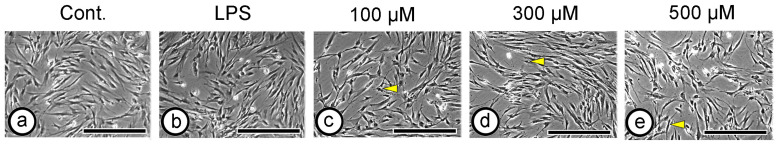
Morphological changes in HDPs after PA incorporation. The morphology was observed under a phase contrast microscope. HDPs showed a spindle or stellate morphology 24 h after the addition of PA. Compared to the control groups (**a**), the morphology of HDPs was not significantly different in the 1 µg/mL of LPS group (**b**). However, the shapes were slightly shrunken in the 100 µM (**c**), 300 µM (**d**) and 500 µM (**e**) of PA groups (yellow arrowheads). Cont.: control group; bars = 50 µm.

HDPs grew at a higher density in the control and LPS groups than in the PA groups (Figure 3a–e). Cell morphology of HDPs showed a spindle or stellate shape 24 h in control and LPS groups (Figure 3a,b). However, the cell morphology revealed a slight shrinkage in 100, 300 and 500 µM PA groups (Figure 3c–e). However, definite characteristics of cell death such as nuclear fragmentation as in apoptosis and increase in cell volume as in necrosis were not detected (Figure 3c–e).

### 3.3. Expression of IL-1β, IL-6 and TNF-α

**Figure 4:**
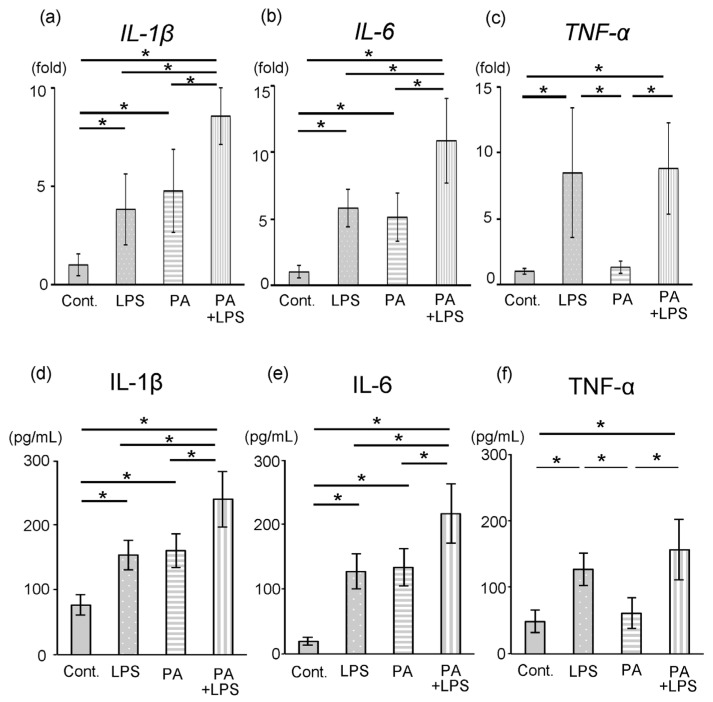
Expression of IL-1β, IL-6 and TNF-α. Data were expressed as means ± standard deviation (SD) (n = 7 each). The mRNA expression of *IL-1β* (**a**), *IL-6* (**b**) and *TNF-α* (**c**) was detected in all groups at 24 h. The concentration of IL-1β (**d**), IL-6 (**e**) and TNF-α (**f**) was measured at 24 h. Cont.: control group; * statistical significance, *p* < 0.05.

The qPCR was performed to confirm the expression of the inflammatory cytokine genes *IL-1β*, *IL-6* and *TNF-α* in all groups (n = 7). Based on the results of cell viability and morphology, we employed 300 µM of PA for adding to the culture medium. *IL-1β* and *IL-6* expression levels were significantly higher in the LPS groups than in the control groups (3.8-fold and 5.8-fold, respectively; *p* < 0.05). The mRNA expression of *IL-1β* and *IL-6* in the PA groups was significantly higher than that in the control group after 24 h (4.9-fold and 5.1-fold, respectively; *p* < 0.05) (Figure 4a,b). The expression level of *IL-1β* and *IL-6* in the LPS groups was the same as that in the PA groups (Figure 4a,b). Although *TNF-α* expression level in LPS group was significantly higher in the control group (7.7-fold; *p* < 0.05), the expression level in the PA group was the same as that in the control group (Figure 4c). The mRNA expression of *IL-1β* and *IL-6* was significantly higher in both the LPS and PA groups than that in the LPS or PA groups (*p* < 0.05) (Figure 4a,b), while the *TNF-α* expression level in the LPS and PA groups were the same as that in the LPS group (Figure 4c).

Likewise, the concentration of IL-1β and IL-6 was significantly higher in the both LPS and PA groups than that in the LPS or PA groups (*p* < 0.05) (Figure 4d,e), while the TNF-α expression levels in LPS and PA groups were the same as that in the LPS group (Figure 4f).

### 3.4. Expression of nlrp3, Caspase-1, Caspase-4 and Gasdermin d

**Figure 5:**
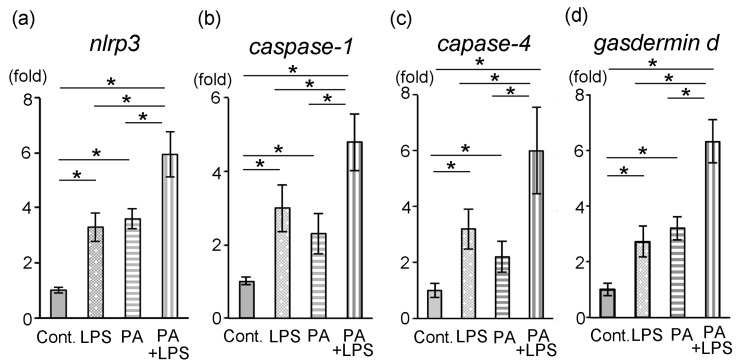
Expression of pyroptosis-related genes. Data were expressed as means ± standard deviation (SD) (n = 5 each). (**a**) *nlrp3*, (**b**) *caspase-1*, (**c**) *caspase-4* and (**d**) *gasdermin d* in the Cont., LPS, PA, and PA + LPS groups (*p* < 0.05). Cont.: control; * statistical significance (*p* < 0.05).

The expression of *nlrp3*, *caspase-1*, *caspase-4* and *gasdermin d* mRNAs was confirmed to understand the relevance of PA-induced pyroptosis in HDP (n = 5). The mRNA expression of *nlrp3*, *caspase-1*, *caspase-4* and *gasdermin d* was significantly higher in the LPS or PA groups than in the control group after 24 h (Figure 5a–d; *p* < 0.05).

## 4. Discussion

We investigated the expression of inflammatory cytokines in response to the addition of PA to cultured HDPs. We previously reported that HDPs expressed inflammatory cytokines, *IL-1β* and *IL-6* [35]. In the present study, we showed that HDPs expressed *IL-1β* and *IL-6* after the addition of PA, while an obvious increase in *TNF-α* was not seen. An increase in inflammatory cytokines induced with PA may be involved in the pulpitis of patients with hyperlipidemia or diabetes. Interestingly, an increase in *TNF-α* was not seen by PA alone, although that was seen by both LPS and PA stimuli as shown in this study and the previous study [36]. The results suggest that PA would not induce *TNF-α* expression.

In the present study, observations of HDP morphology revealed a slight shrinkage without definite apoptotic and necrotic findings in the various PA concentration groups. Furthermore, cell viability showed significant differences between the control group and 300 µM group, and between the control group and 500 µM group. PA has been shown to induce necrosis in RAW264.7 cells (macrophage-like cells) [37], as well as apoptosis via the TLR4 pathway in vascular smooth muscle cells [38]. The results suggest that the experimental conditions used in this study affect cell viability or morphology, but time point (24 h) or concentration may not be enough to induce cell death. Furthermore, we employed the same PA concentration according to the method by Sun et al. who used periodontal ligament fibroblast [24]. We have previously reported that dental pulp cells can withstand various conditions such as hypoxia [39], low glucose [40] and heat stress [41,42]. Dental pulp cells would be able to withstand the experimental conditions used in this study.

LPS is a strong inducer of pro-inflammatory cytokines, such as IL-1β, IL-6 and TNF-α, via the TLR4 and NF-kB signaling pathways [43]. Recent studies examined the mechanisms underlying the PA-induced inflammatory cytokine production [21,38,44,45]. Although TLR4 is involved in the regulation of PA-induced inflammation, it is not a receptor for PA [46]. Previous studies demonstrated that PA induced the expression of IL-6 by activating the TLR4 signaling pathway [21,38], and the expression of IL-1β by not only up-regulating the expression of TLR2, but also by activating the inflammasome NLRP3 in hepatocytes [47]. In the present study, we showed that PA promoted the expression of IL-1β and IL-6 in HDPs, which is consistent with previous findings on other cell types. Furthermore, higher expression of IL-1β and IL-6 was seen in the PA + LPS group compared to the PA or LPS group in this study. LPS derived from *P. gingivalis* activates both TLR4 and TLR2, and TLR2 activation induces pro-inflammatory cytokines in human odontoblast and dental pulp fibroblast [48]. Moreover, the overactivation of the cytokine-mediated JAK2/STAT3 pathway is involved in the expansion and persistence of inflammation [49]. Our results imply that LPS derived from *P. gingivalis* enhances PA-induced inflammation synergistically via some pathways. However, we did not examine the relationship between PA and TLRs, and further study is needed.

Our results showed that the addition of PA changed the cell morphology and increased the expression of *nlrp3*, *caspase-1*, *caspase-4* and *gasdermin d* that were associated with pyroptosis. Pyroptosis is a kind of inflammatory programmed cell death that is mainly dependent on caspases and gasdermins and classified as regulated cell death. Recently, pyroptosis has been reported to play an important role in obesity- and diabetes-associated inflammation [30]. Furthermore, pyroptosis participates in the pathogenesis of periodontitis, and increased level of pyroptosis markers is found in gingival tissue of diabetes-associated periodontitis [50]. However, the involvement of PA remains unknown about pulpitis. Therefore, we analyzed the changes in pyroptosis-related genes such as *nlrp3*, *caspase-1*, *caspase-4* and *gasdermin d*. Caspase-4 plays an important role in the non-canonical pathway of pyroptosis and may directly recognize intracellular LPS, leading to its self-activation [24]. PA was shown to activate caspase-4, and the caspase-4 cleaved gasdermin d, resulting in the formation of N-terminal fragments that make pores in the cell membrane, causing cell swelling and rupture, and promoting the release of IL-1β [51,52]. In this study, the expression of *NLRP-3*, *caspase-1*, *caspase-4* and *gasdermin d* in the PA group was significantly increased compared to the control group, suggesting that PA promotes the activation of caspase-4, induces the cleavage of gasdermin d, and pyroptosis in the HDP.

Although we could not confirm the morphology of pyroptosis, qPCR data showed the expression of pyroptosis related to mRNA such as *nlrp3*, *caspase-1*, *caspase-4* and *gasdermin d*, suggesting cell death due to PA is pyroptosis in dental pulp cells. If we changed the time points or concentration of PA, we would confirm the cell morphology of pyroptosis. This is the limitation of this study and what further studies need to clarify.

## 5. Conclusions

The relevance between diabetes mellitus and dental pulp has been shown and the dental pulp of patients with diabetes is in a state of inflammation because of nerve damage and local microcirculation breakdown, finally leading to pulp necrosis [53,54,55]. However, detailed relevance between hyperlipidemia and dental pulp inflammation remains elusive at present. In this study, we focused on the PA, assuming hyperlipidemia or diabetes mellitus, and demonstrated that PA induced the expression of inflammatory cytokines and pyroptosis. We showed the decline in cell viability, shrink shape of the cells, and increase in the expression of inflammatory cytokines and pyroptosis-related genes under PA conditions in a human dental pulp cell line. The results of this study suggest PA-induced inflammatory and pyroptotic responses in human dental pulp cells and may potentially contribute to pulp lesions such as inflammation and dystrophic calcification in patients with hyperlipidemia or diabetes mellitus. The results would highlight the relevance between pulp lesions and systemic diseases such as hyperlipidemia and diabetes mellitus. Dentists need to consider pulp inflammation condition and calcification when they examine the patient with hyperlipidemia or diabetes mellitus.

## Data Availability

The data presented in this publication are available on request from the corresponding author. The data are not publicly available due to privacy restrictions.

## Abbreviations

The following abbreviations are used in this manuscript:

PA	Palmitic acid
FFA	Free fatty acid
TLR	Toll-like receptor
LPS	Lipopolysaccharide
MEM	Minimum essential medium
HDP	Human dental pulp cell
